# Crack propagation and fracture in silicon wafers under thermal stress

**DOI:** 10.1107/S0021889813003695

**Published:** 2013-06-07

**Authors:** Andreas Danilewsky, Jochen Wittge, Konstantin Kiefl, David Allen, Patrick McNally, Jorge Garagorri, M. Reyes Elizalde, Tilo Baumbach, Brian K. Tanner

**Affiliations:** a Kristallographie, Institut für Geowissenschaften, University Freiburg, D-79104, Germany; b Research Institute for Networks and Communications Engineering, Dublin City University, Dublin, Ireland; c CEIT and Tecnun, University of Navarra, San Sebastian, Spain; d Institut für Photonen­for­schung und Synchrotronstrahlung, Karlsruhe Institute of Technology, Karlsruhe, Germany; e Department of Physics, University of Durham, South Road, Durham DH1 3LE, England

**Keywords:** microcracks, silicon wafer fracture, *in situ* observations, X-ray diffraction imaging

## Abstract

The behaviour of microcracks in silicon during thermal annealing has been studied using *in situ* X-ray diffraction imaging. Initial cracks are produced with an indenter at the edge of a conventional Si wafer, which was heated under temperature gradients to produce thermal stress. At temperatures where Si is still in the brittle regime, the strain may accumulate if a microcrack is pinned. If a critical value is exceeded either a new or a longer crack will be formed, which results with high probability in wafer breakage. The strain reduces most efficiently by forming (*hhl*) or (*hkl*) crack planes of high energy instead of the expected low-energy cleavage planes like {111}. Dangerous cracks, which become active during heat treatment and may shatter the whole wafer, can be identified from diffraction images simply by measuring the geometrical dimensions of the strain-related contrast around the crack tip. Once the plastic regime at higher temperature is reached, strain is reduced by generating dislocation loops and slip bands and no wafer breakage occurs. There is only a small temperature window within which crack propagation is possible during rapid annealing.

## Abstract 2

The microcrack propagation and cleavage behaviour in silicon wafers during thermal annealing has been studied by *in situ* X-ray diffraction imaging (topography).

## 1. Introduction

During semiconductor manufacture, wafer preparation and handling can result in damage in the form of microcracks at the edges of the wafers (Cook, 2006 ▶). While most of the damage appears to be harmless, under some conditions edge-damaged wafers can catastrophically fracture during rapid thermal annealing (RTA) (Chen *et al.*, 2009 ▶, 2010 ▶; Tanner *et al.*, 2012 ▶). There is also evidence that, under certain thermal treatments, slip can be induced at the bevel edge or wafer extremity (Tanner *et al.*, 2011 ▶) and this may extend across a substantial fraction of the wafer (Garagorri *et al.*, 2012 ▶). Sig­nificant effects on bipolar device yields have been found when thermally induced dislocations are present (Schwuttke, 1998 ▶). The microcracks, as well as the dislocations forming slip bands, can be characterized by X-ray diffraction imaging (XRDI), also known as X-ray topography (Bowen & Tanner, 2006 ▶).

In previous studies using *in situ* XRDI a model for thermal slip formation from microcracks is proposed (Wittge *et al.*, 2010*a* ▶). However, under certain conditions the microcrack may increase dramatically in length, finally cleaving the wafer into two or more pieces. The preferred cleavage of silicon is along {111} and {110} planes, but in the presence of high stress or strain gradients, for example during fast annealing procedures, fracture on higher-order planes is also observed, as multiple factors influence the direction of crack propagation. A number of theoretical studies based on numerical simulations and/or experiments applying mechanical stress mainly at room temperature confirm the deflection from planes with higher energy into {111} as the energetically preferred cleavage plane with the crack tip propagating into 〈110〉 or 〈112〉 directions (*e.g.* Kermode *et al.*, 2008 ▶; Sherman, 2006 ▶). In Si(100) wafers the formation of {110} crack planes will again minimize the total energy of the crack because the cleavage plane perpendicular to the (100) wafer faces results in a smaller crack surface area than any other inclined cleavage plane (Sherman, 2006 ▶).

To generate, in a controlled manner, defects similar to those induced by handling, well defined microcracks were generated in Si(100) wafers with a nanoindentation method close to the edges of 20 × 20 mm samples. The defects and the associated strain have been studied *in situ* at high temperature with white-beam X-ray diffraction imaging at the synchrotron light source ANKA, at the Institute for Photon Science and Syn­chro­tron Radiation, Karlsruhe Institute of Technology, Germany.

In recent papers we have described how, above the brittle to plastic transition temperature, the strain around defects is reduced by dislocation loop and slip band formation (Wittge *et al.*, 2010*b* ▶; Danilewsky, Wittge, Hess, Cröll, Allen *et al.*, 2011 ▶). In the present study we show that, during *in situ* heating in a mirror furnace, the strain from thermal stress may accumulate if the opening of a microcrack is impeded. If a critical value is exceeded either a new or a longer crack will be formed, resulting in wafer breakage.

## 2. Cleavage planes and crack propagation in Si

Si crystallizes in the diamond structure and shows a perfect cleavage along {111} and {110}. This is different from the cleavage of diamond itself. In addition to the cleavage along the {111} planes, a micro cleavage along {110} and between {111} and {100} in the 〈110〉 zones (Goryunova, 1965 ▶) can be observed. Looking along a (110) projection of the crystal lattice, it becomes obvious that the bonds of the primary cleavage planes {111} are perpendicular to the direction of the cleavage. The atomic bonds of the secondary cleavage plane {110} are inclined to the direction of the cleavage. Together with the (100) surface, both cleavage planes, primary (111) and secondary (110), belong to the same crystallographic zone. There is straight cleavage along the 〈110〉 directions and the possibility of change between the primary and the secondary cleavage plane during cleavage itself (Cook, 2006 ▶). The relative numbers of bonds per unit area crossing the planes, which have to be broken during cleavage, define the surface energy γ_*hkl*_. For the {111}, {110}, {112} and {100} planes in the diamond structure, this number is in the ratio 1:(3/2)^1/2^:2^1/2^:3^1/2^ and the related energy to form the new surfaces increases (Cook, 2006 ▶). As a consequence, the energy needed to cleave along the respective planes by breaking the bonds and forming two surfaces increases in this order. For a critical crack driving force (fracture energy) at least two times the surface energy γ_*hkl*_ is needed because two new surfaces are to be formed (Ebrahimi & Kalwani, 1999 ▶). Tanaka *et al*. (2006 ▶) discuss this result in terms of fracture toughness, which is proportional to the surface energies. They also show that the values of surface energy for {11*l*} cleavage planes parallel to 〈110〉 directions are between the values for {112} and the highest possible for {100} planes and always higher than for {111} planes: 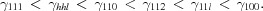
Alternatively, we note that, if the crystal breaks as a consequence of applied stress, the energy annihilated scales in this manner. This was demonstrated by Sherman (2006 ▶) for (110) and (111) planes, where from a pre-crack with average propagation energy and velocity a crack starts propagating in the (110) plane to deflect in the lower-energy (111) plane.

The different binding energies between lattice planes result in an anisotropy of the mechanical and elastic properties, which are commonly described by Young’s modulus *E* and the shear modulus *G*, respectively. *E* is the ratio of tensile stress to strain (Hopcroft *et al.*, 2010 ▶), which describes how the stiffness properties vary with direction in the crystal. Young’s moduli for various lattice directions range between the highest value of 188 GPa for the 〈111〉 directions and 130 GPa for the 〈100〉 directions. If the surface energy increases, Young’s modulus decreases (Tanaka *et al.*, 2006 ▶). Again it can be concluded that cleavage should preferentially occur along {111} planes. However, owing to conchoidal fracture the cleavage plane may change to higher-indexed planes because of the influence of external stress. During fracture, more energy can be annihilated by forming higher indexed cleavage planes of higher energy.

*G* is the ratio of shear stress to strain, which describes how the stiffness properties vary between the directions in the crystal. The shear moduli between various lattice directions of Si range, for example, in {100} planes from 50.9 GPa (by twisting between the 〈110〉 directions, which is the lowest value) to 79.4 GPa (between the 〈100〉 directions, which is the highest value) (Hopcroft *et al.*, 2010 ▶). The minimum stress σ that is needed to form a crack is given in equation (1) by the Griffith criterion (Griffith, 1921 ▶; Hirth & Lothe, 1982 ▶):
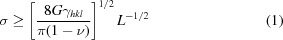
with the shear modulus *G*, Poisson’s ratio ν and the length of the crack *L*.

Once a crack is formed, residual long-range strain, especially around its tip, can be revealed by XRDI. In a simple model of image formation based on a semi-kinematical formalism (Tanner *et al.*, 2012 ▶) the formation of the so-called direct defect image arises from regions around the defect where the effective misorientation δ(Δθ) associated with the deformation of the lattice planes is equal to or larger than the full width at half-maximum of the perfect crystal reflection range (Darwin width) δω (Bowen & Tanner, 2006 ▶). Therefore the XRDI contrast width is directly related to the back stress on the crack tip, which may be modelled as an edge dislocation pile-up. The result is a super-edge dislocation *Nb* where the crack width *d*_0_ is a multiple *N* of the magnitude *b* of the Burgers vector **b** of an edge dislocation.

Using again the Griffith criterion (Griffith, 1921 ▶) and rewriting equation (1), we have for the critical stress σ at which a crack propagates (Tanner *et al.*, 2012 ▶)
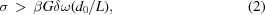
where β is a numerical parameter of value between 5 and 10 and *d*_0_ is the X-ray image width at the crack tip. The stress in this case is the thermal stress inside the mirror furnace, given by vertical and horizontal temperature gradients. The critical parameter κ_c_ to measure in the XRDI image is therefore the crack length *L* divided by X-ray image width *d*_0_ at the crack tip: 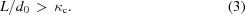



Cracks with a small length to width ratio are benign; those with a small image width at the crack tip and long length are highly dangerous. The value of κ_c_ for crack propagation in an RTA sequence is determined by the thermal stress across the sample and depends critically on the heater geometry, as well as the heating and cooling rates (Tanner *et al.*, 2012 ▶).

## 3. Experimental

Experiments were conducted on integrated circuit quality, 200 mm (‘8 inch’) diameter, dislocation-free silicon wafers cut within 0.2° of the (100) orientation. The double-side-polished p-type wafers had resistivity below 10 Ω mm and were of thickness 725 (25) µm. No edge defects were visible either under optical inspection or in XRDI images of the as-received wafers, which had been packed and shipped in standard cassettes. Indentation was performed with a Vickers tip (Garagorri *et al.*, 2010 ▶) in order to produce well defined amounts of damage at specific locations adjacent to the bevel edges. For the *in situ* RTA experiments, samples of size ∼20 × ∼20 mm were cut from wafers subjected to loads of up to 50 N. The strain fields associated with the damage and those from slip dislocations were revealed by XRDI, in white-beam transmission mode, at the TOPO-TOMO beamline at the ANKA synchrotron radiation facility, using an indirect digital detector system (Danilewsky, Wittge, Hess, Cröll, Rack *et al.*, 2011 ▶).

A double ellipsoidal mirror furnace (Eyer *et al.*, 1979 ▶; Danilewsky, Wittge, Hess, Cröll, Allen *et al.*, 2011 ▶) capable of achieving 1473 K was used for the heating. Owing to the focusing of the light from the two 450 W halogen lamps into a small volume, there are high thermal gradients across the sample, namely 18 K mm^−1^ in the vertical and 8 K mm^−1^ in the horizontal directions, which correspond to the 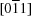 and [011] directions, respectively, of the sample shown in Figs. 2–4.

**Figure 1:**
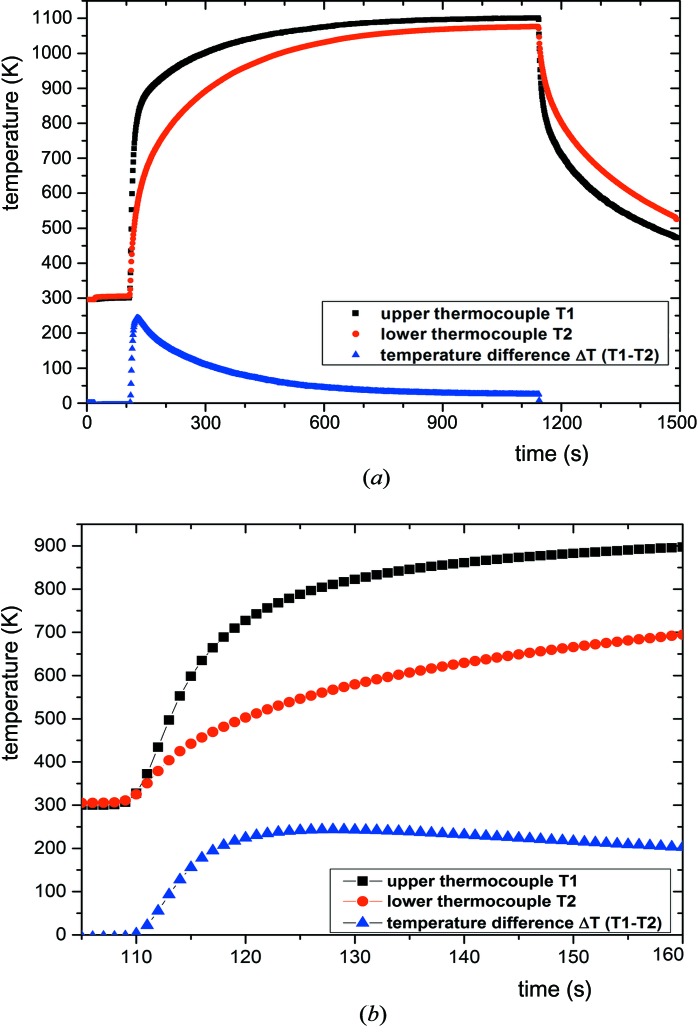
(*a*) Full timeline of the first heating of wafer 0110. The temperature was measured every second. (*b*) Beginning of heating with higher time resolution. The crack opens at a temperature of 598 K, *i.e.* for a temperature difference Δ*T* of 155 K between the two thermocouples. The side crack was generated between 893 and 895 K, with Δ*T* = 204 K at about 155–160 s.

The temperature was measured with two thermocouples in contact with the back side of the sample situated near the centre and 6 mm below. Fig. 1 ▶(*a*) shows the typical heating profile used for the first heating sequence and Fig. 1 ▶(*b*) the first 50 s of the heating with higher time resolution. The applied RTA heating rate is the same as in plateau annealing but much slower than in flash annealing methods. The data relate to the experiment that corresponds to the movie available as supplementary material^1^ (from which the series of single topographs shown in Fig. 3 below have been taken). A second, similar, heating sequence was applied to observe the formation of slip systems at different positions along the cracks.

Images can be recorded with an integration time of 0.1–1 s on a CCD camera (Danilewsky, Wittge, Hess, Cröll, Rack *et al.*, 2011 ▶) optically coupled to a macroscope and with an Lu_3_Al_5_O_12_ scintillator. The effective pixel size of the detection system was 2.5 × 2.5 µm. For the movie of the crack development, an X-ray image was recorded every 0.72 s, set by the timestamp on the images. The integration time per image was 0.25 s. The remainder of the time period (0.47 s) was required for readout and storage time. The single frames from 0001 to 0*xyz* were assembled into a high-speed *in situ* movie of the crack and slip formation during heating and plateau annealing. After a second heating cycle the visible part of the cracks was analysed by Nomarski interference contrast (NIC) and infrared transmission microscopy.

## 4. Results and discussion

**Figure 2:**
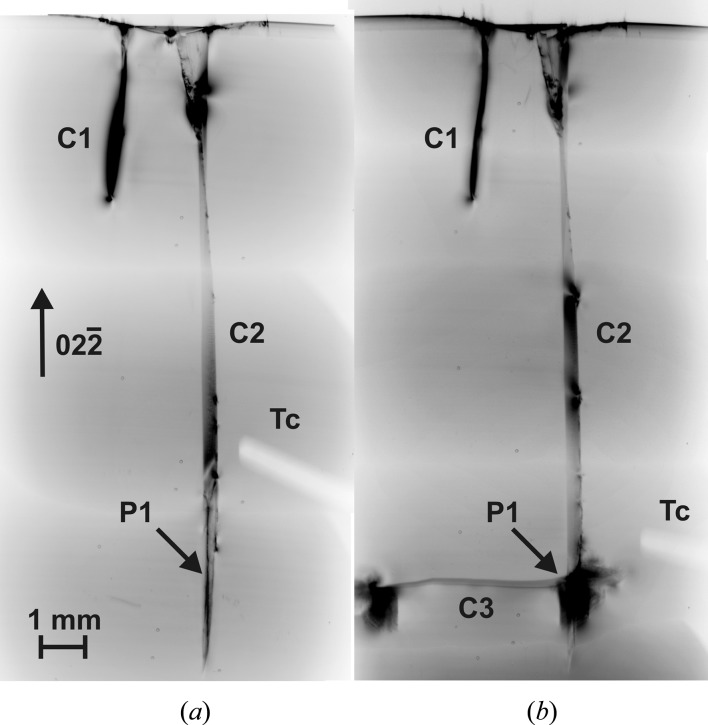
Transmission topographs of two cracks introduced from a 50 N indentation at the wafer edge, 90° from the notch: Tc indicates the shadow of a thermocouple, P1 the position where crack C3 originates (room temperature, view from the back side through the sample). (*a*) Before the first heating sequence. The left crack C1 is short and shows a substantial strain field at the tip of the crack for which κ = 20. The more dangerous crack C2 is almost four times longer and has a sharp contrast at the tip; no strain field is visible which results in κ = 167. (*b*) After the first heat treatment. The new horizontal crack C3 is generated. Around the crack tips dense dislocations and slip bands were produced, visible from the black contrasts.

Subcritical and critical cracks introduced by near-edge indentation are shown in the large area transmission topograph of Fig. 2 ▶(*a*). The shorter crack C1 on the left has a low κ value (*L*/*d*_0_ = 20), which is below κ_c_ for all our experiments. It does not propagate throughout the experimental cycle and the X-ray image width decreases during the heating sequences (Fig. 2 ▶ *b*). On the right is a longer crack C2 with a high κ value (*L*/*d*_0_ = 167), which exceeds the κ_c_ value associated with the heating and cooling sequence shown in Fig. 1 ▶. As shown in Fig. 2 ▶(*b*), crack C2 does propagate.

The wafer’s edge is heavily damaged around the indent. The first 2 mm of the crack C2 show orientation contrast from tilted platelets cleaved by the 50 N load. By measuring the average width of the X-ray image associated with the length of crack C2 into 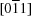 and comparing it with the calculated theoretical values for the crack widths from the different diffraction planes, it can be concluded that the crack runs from different (11*l*) planes into a predominantly 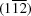 plane. Some irregularities indicate conchoidal fracture. Since the critical crack lengths even for the narrowest images are greater than the wafer thickness, the cracks penetrate through to both sides of the wafer, except the last 2.3 mm of the tip. This ending part of the tip is not visible either at the surface by NIC or in the bulk by infrared transmission light and therefore cannot be indexed, but {110} and {111} can be excluded. We then expected them to be visible in reflection XRDI as well as in transmission, and careful X-ray experiments on both sides of the wafer confirmed this to be the case. The crack C3 in Fig. 2 ▶(*b*) appeared at relatively low temperature in the brittle regime of silicon in the perpendicular 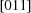 direction (compare Fig. 1 ▶ *b*). This crack starts again through complex high-indexed planes capable of consuming much of the strain energy. Then it deflects for a long distance into the low-energy 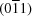 plane until it ends in another more complicated plane which cannot be indexed any more.

**Figure 3:**
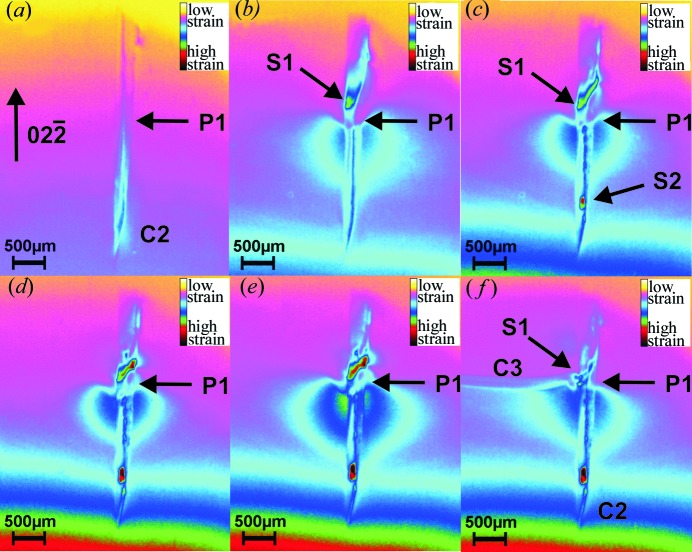
False-coloured X-ray diffraction images around the tip of crack C2 (detail from Fig. 2 ▶ *a*). (*a*) Image 0037 at room temperature before heating of the crack C2. P1 marks the position where the opening of the crack is impeded. (*b*) Image 0045 at ∼598 K (Δ*T* ≃ 155 K). The image shows the opening crack which produces a huge strain field below P1 and a small one S1 directly above. (*c*) Image 0049 at 689 K shows increasing strain fields, the upper one into the [00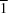] direction. A small-sized strained area S2 builds up near the tip. (*d*) Image 0060 at 788 K shows increasing strain at S1 above P1. (*e*) Image 0104 at 893 K. The strain fields below and above P1 have both increased in size and intensity. The thermal stress exceeds 24.8 MPa. (*f*) Image 0105 at 895 K. The crack C3 has developed to the left into the [011] direction in less than 0.72 s and the strain fields have significantly decreased in size and intensity.

**Figure 4:**
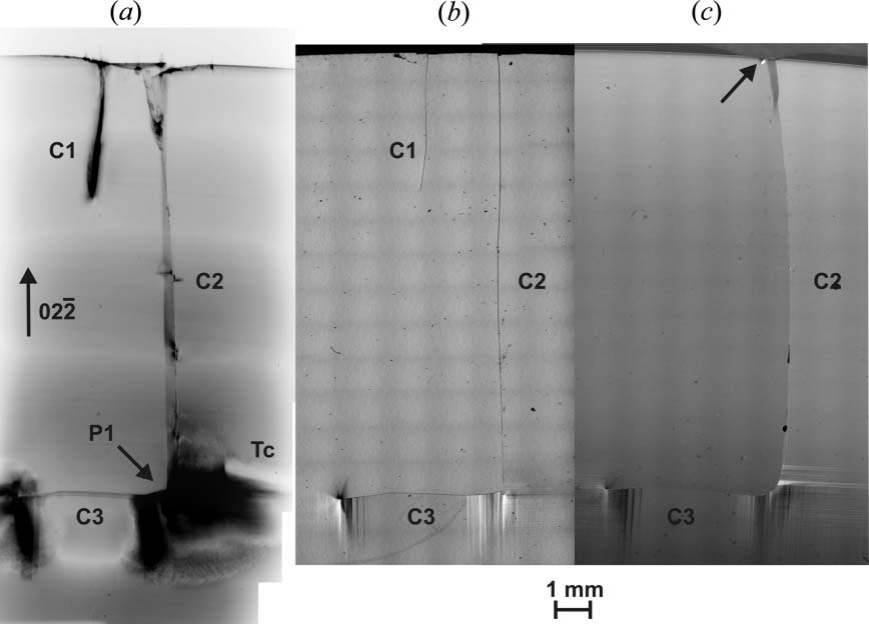
(*a*) X-ray diffraction transmission-mode image of the crack after the second heating cycle viewed from the back side through the sample, taken at room temperature. Around the tip of the initial crack and the lateral crack tip, many dislocations and slip bands have been generated. (*b*) Interference contrast microscopy image from the back side with cracks C1, C2 and C3 visible. Surface steps around the tips of cracks C2 and C3 are traces of the slip bands in Fig. 4 ▶(*a*). (*c*) Interference contrast microscopy image of the front side of the same sample (mirrored). Cracks C2 and C3 are visible and the surfaces steps correspond to the slip bands in Fig. 4 ▶(*a*). The arrow on top indicates the position of indentation.

Fig. 3 ▶ shows a series of six topographs around the tip of the crack C2. These images were taken from a movie of the whole heating process, then image processed to improve the contrast and converted into false colours for improved visibility of the contrast related to the strain fields. In Fig. 3 ▶(*a*), at room temperature before heating, a higher strain is visible at the crack tip, indicated by the blue colours. This part of the crack below position P1 is inside the bulk of the wafer and not visible at the surfaces (compare Figs. 4 ▶ *b* and 4 ▶ *c*). Directly after heating, crack C2 opens and stops at position P1, about 2.3 mm away from the visible sharp crack tip (Fig. 3 ▶ *b*), which can be seen in images 0038–0048 on the video of the crack development. Obviously the opening of the crack appears only for this part of the crack where the cleavage plane makes the connection between the front and back side of the wafer. In the part of the crack that remains invisible below the surfaces, a long internal boundary line between the crack and the undisturbed crystal with a length of at least 4.6 mm results. Much more energy would be needed to elongate such a buried crack than the shortest direct connection, which would be, for example, a straight 〈110〉 line with a length of only about 1.0 mm between the wafers surfaces. Between images 0049 and 0104 (corresponding to a temperature rise from 635 to 893 K) the opened crack remains at this position. During this period, for which the crack remains sessile, a substantial long-range strain field below P1 gradually builds up, visible as the dark-blue colours in Figs. 3 ▶(*b*)–3(*e*). Above P1 in parallel a much higher strain piles up in a very small volume S1, like a hot spot and into the 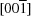 direction, visible as the green–red colours. Another hot spot of strain S2 near the tip remains without consequences during the experiment.

In the next frame, number 0105 (shown in Fig. 3 ▶ *f*), the crack C3 has developed to the left in less than 0.72 s in the [011] direction using first a number of inclined {hh*l*} planes to deflect into the (0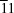) plane perpendicular to the (100) sample surface. It stopped at a point ∼5.4 mm away from P1 after another deflection into higher-energy planes of the type {*hkl*}, deviating from the [011] direction. The duration for which crack C2 remained at position P1, calculated by summing the number of images, is around 40 s as the temperature increases from 598 to 893 K (Fig. 3 ▶ *e*). It becomes obvious that the applied thermal stress was still not high enough to elongate the crack C2 into the direction of the highest gradient and the [011] direction is the more attractive one.

Comparing the strain field in false colours below the crack front, it is clear that most of the strain energy in the lattice was released as the crack bent rapidly and increased in length towards the left hand side in the X-ray image. The spatial extent of the strain field in the lattice decreased on the left hand side as well as on the right hand side of the crack C2. The lower decrease on the left hand side is most likely due to the deformation of the strain field during the development of the side crack.

Using equation (1) and tabulated values of γ, *G* and ν, from the measured length of the side crack C3 of 5.4 (2) mm we deduce that the minimum stress in the crystal lattice for spontaneous growth of the crack has to be larger than 24.8 (6) MPa.

Not all the associated energy was released, because after the development of the crack there was still strain energy left in the crystal lattice, which can be seen in the remaining long-range strain fields beside and below the crack tips C2 and C3. Upon further heating, the length of the crack did not increase, but in the region of the strain fields of the stationary point at P1 and around the crack tip itself dislocations and slip bands are generated (Fig. 2 ▶ *b*). The onset of the slip formation starts with image 0540 in the movie and it occurs at a temperature of ∼1056 K, about 364 s after the start of the heating cycle.

We have shown previously that the dislocation velocity follows an Arrhenius behaviour with an activation energy of 2.1 (2) eV (Tanner *et al.*, 2011 ▶). There is no abrupt transition between brittle and plastic regimes, but the value of 1056 K is about the point at which dislocation motion becomes detectable in XRDI (Fig. 8 in Tanner *et al.*, 2011 ▶). The image of the primary sharp crack tip of C2 became obscured upon further heating by dislocations and slip bands (Figs. 2 ▶ *b* and 4 ▶ *a*), which appear to have been introduced from the strain field around the stationary point (P1) of the developed crack. It is also to be noted that dislocations and slip bands were generated only on the side at which a strain field was present. Comparing the images in both Fig. 2 ▶(*b*) and Fig. 4 ▶(*a*) with Fig. 2 ▶(*a*) before the initial heating, it is observed from the top part of the X-ray images that during the heating process the right side of the crack shifted downwards. We note that, for a crack of this κ value in a 200 mm wafer taken through the cycle in Fig. 1 ▶ within our commercial RTA apparatus, catastrophic fracture would almost certainly have occurred (Tanner *et al.*, 2012 ▶). The small sample has not fractured in the mirror furnace because the stress on cooling was less, owing to the very different geometrical conditions and gradients that define the transient areas from compressive to tensile strain (Garagorri *et al.*, 2012 ▶).

On taking the sample around the heating cycle for a second time, we observed no further changes in the crack position (Fig. 4 ▶ *a*). Obviously the residual stress is not enough to produce another cracking. However, there was further development in the dislocation configuration at the tip of the lateral crack and at the point of branching. The extent of the high dislocation density in Fig. 4 ▶(*a*) is roughly twice that in Fig. 2 ▶(*b*), which is consistent with the sample spending twice the length of time in the temperature region where significant plastic deformation occurred.

NIC (Figs. 4 ▶ *b* and 4 ▶ *c*) resolves surface steps that are the traces of slip bands (Danilewsky, Wittge, Hess, Cröll, Allen *et al.*, 2011 ▶) on the front and back surfaces of the sample, and we note that they correspond well with the X-ray images. As already mentioned, not all parts of the cracks can be seen at the wafer’s surfaces with light microscopy. Only on the back side of the sample are all three cracks visible (Fig. 4 ▶ *b*). On the front side the indentation from which both initial cracks originate is indicated by an arrow, but the crack C1 is completely invisible (Fig. 4 ▶ *c*). The crack C2 appears macroscopically more curved on the front side and 2.3 mm shorter than the value obtained by topography. The path of crack C2 on the inclined cleavage plane inside the sample is clearly different on the front and back sides. Therefore it cannot consist of one single and smooth 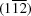 cleavage plane as concluded from the diffraction image in Fig. 2 ▶, but deflects into various planes not only from the indent on top down to the tip but also in between the front and back sides of the sample. The straight [0
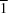
1]-oriented path visible on the back side (Fig. 4 ▶ *b*) as well as in the diffraction images [Figs. 2 ▶ and 4 ▶(*a*)] indicates that the crack surface oriented to this side is predominantly (011).

The further complexity of crack planes becomes evident at position P1, where crack C3 starts on the back side nearly perpendicular in [011] but is more curved on the front side using a number of inclined high-energy-consuming (*hhl*) and (*hkl*) planes, which cannot be indexed any more. Finally it deflects into the (0
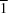
1) plane perpendicular to the (100) surface, which opens the smallest area of the cleavage plane between the two sides of the sample and results in a smaller total surface energy than any other inclined cleavage plane in the same position would have (Sherman, 2006 ▶).

**Figure 5:**
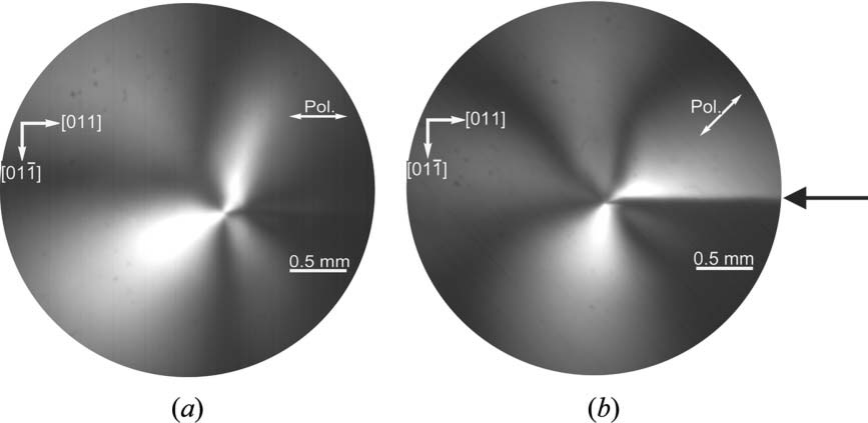
Polarized infrared images showing the strain around the tip of crack C3 after the second heating (black arrow indicates crack position). The asymmetric lobes indicate the inclined 60° dislocations to be the origin of the strain. (*a*) Polars parallel and perpendicular to crack direction. (*b*) Polars at 45° to crack direction.

Polarized infrared images of the strain fields around the tip of crack C3 after the second heating sequence (Fig. 5 ▶) show a long-range strain field consistent with the extent of the high-dislocation-density region. This strain field shows a highly asymmetric four-lobe contrast, somewhat similar to that expected of a super-edge dislocation. However, the strongest lobe lies at approximately 120° to the crack direction when the crossed polars are parallel (Fig. 5 ▶ *a*) and perpendicular to the crack direction, and at 0/90° to it when the polars are at 45° to the line direction (Fig. 5 ▶ *b*). A contrast of ±45° for parallel polars and 0/90° for polars in the 45° position would be expected for a super-edge dislocation (Tanner & Fathers, 1974 ▶) running perpendicular to the wafer surface, but with the Burgers vector in the wafer plane inclined at 45° to the crack length. However, the simple model of the crack tip has the super-Burgers vector at 90° to the crack, inconsistent with the results of Fig. 5 ▶. It is evident from Fig. 4 ▶(*a*) that substantial slip, assumed to consist of a high number of 60° and screw dislocations on inclined {111} planes (Danilewsky, Wittge, Hess, Cröll, Allen *et al.*, 2011 ▶), has occurred in the vicinity of the crack tip and has modified the overall strain field. In the same way as described by Booyens & Basson (1980 ▶) for the sphalerite structure, only the edge part of the Burgers vector of the 60° dislocations contributes to the contrast, which explains the asymmetry of the lobes in Fig. 5 ▶. From this it can be concluded that the residual strain in the polarized infrared images after the second heating is related to the dislocations and not to the crack.

## 5. Conclusions

The changes in behaviour of well defined, artificially induced cracks at the edge of an Si wafer have been observed *in situ* by X-ray diffraction imaging during heating in a mirror furnace. Very different behaviour is observed during heating from cracks that show different X-ray contrast. This further strengthens support for the model in which a critical value κ_c_ of the parameter κ, measured directly from the X-ray image as the aspect ratio (*L*/*d*_0_) of the crack tip, can be used to determine whether the crack will or will not propagate under stress (Tanner *et al.*, 2012 ▶). The measured length to X-ray image width ratio *L*/*d*_0_ from the diffraction images of the tip of crack C2 is around eight times higher than that for crack C1. Whereas the strain field around C1 reduces in size as a result of the annealing process, the crack C2 opens and more strain is produced in the surrounding and previously undisturbed crystal lattice, especially if the area below the crack is pinned. Finally by exceeding a critical value of 24.8 MPa at *T* = 895 K, the crack C2 elongates and a new perpendicular crack C3 develops. This process takes place extremely rapidly, within a single frame, and no details of the propagation of the new crack could be determined. Taking the average speed of a crack in a brittle material to be 1500 m s^−1^ (Sherman, 2006 ▶), a frame rate of a digital camera system of about 280 000 frames per second would be needed to resolve the formation of the 5.4 × 10^−3^ m long crack C3 in Fig. 2 ▶(*b*), whereas to date high-speed systems are only available up to 105 000 frames per second (Garcia-Moreno *et al.*, 2011 ▶).

The X-ray images compared with light microscopy images show that the cracks do not start, or propagate always, on the low-energy planes and that, even under apparently ideal conditions, significant deviation towards high-energy planes takes place. In a small sample the strain energy is released to a great extent and the crack front is pinned. This is especially true if the energy is not high enough to overcome the fracture stiffness along the extended borderline between open crack surface and undisturbed crystal, in which case the crack runs over a long distance inside the bulk of the wafer and does not follow the shortest connection between the wafer surfaces. The absorbed energy may unload if a critical value is reached by using first {*hhl*} or {*hkl*} planes and then deflecting into a {110} plane perpendicular to the (100) wafer faces, because this generates the smallest free surface area with a minimum of surface energy (Sherman, 2006 ▶).

For a small sample in the mirror furnace, the stress in the brittle regime is not large enough to elongate the crack at low temperatures. When the temperature of the sample exceeds the brittle to ductile transition, dislocation loops and slip bands form to relieve the crack energy (Wittge *et al.*, 2010*a* ▶; Danilewsky, Wittge, Hess, Cröll, Allen *et al.*, 2011 ▶), effectively blunting the crack tip and preventing elongation of the crack. Only in a very small temperature window does crack propagation occur. Once strain is reduced in the plastic regime by forming dislocations and slip bands, not enough energy can be collected for crack formation. The stress produces more and more dislocations and slips and is no longer dangerous for wafer breakage.

While the heating rate in our experiments is slow compared with that associated with very short flash annealing processes, our *ex situ* RTA experiments show that wafer fracture always occurs during cooling, when the centre of the wafer is hotter than the perimeter (Tanner *et al.*, 2012 ▶). Although finite element modelling is necessary to determine whether fracture is predicted in specific conditions, we may expect our conclusions to be relevant to flash annealing as well as other RTA processes.

For a large wafer similar behaviour has to be expected but with smaller critical values, because of the less efficient strain release as a function of the longer distances to the wafer edges.

## Supplementary Material

Movie showing X-ray diffraction images around a crack tip during heating. DOI: 10.1107/S0021889813003695/xz5004sup1.wmv

## Footnotes

1 The movie discussed in this paper is available from the IUCr electronic archives (Reference: XZ5004). Services for accessing this material are described at the back of the journal.
